# Individualized treatment for pediatric intracranial aneurysms in China: case series and literature review

**DOI:** 10.3389/fsurg.2026.1838917

**Published:** 2026-06-11

**Authors:** Guohong Song, Haihui Jia, He Tong, Hua Xu, Changyi Hu, Bo Li, Hao Chen

**Affiliations:** Department of Neurosurgery, Affiliated Hospital of Jining Medical University, Jining, Shan Dong, China

**Keywords:** endovascular embolization, individualized treatment, microsurgical clipping, pediatric intracranial aneurysms, rupured aneurysm

## Abstract

**Objective:**

Pediatric intracranial aneurysms are rare, accounting for a small proportion of all aneurysms, yet they often present with acute onset, rapid deterioration, and a high risk of rupture. Diagnostic and therapeutic standards remain inconsistent due to limited evidence.

**Methods:**

We retrospectively reviewed four pediatric patients (≤14 years) with ruptured intracranial aneurysms treated at our center from January 1, 2021 to December 31, 2025. Clinical presentation, imaging findings, treatment selection, and postoperative outcomes were analyzed. Functional outcomes were assessed using the modified Rankin Scale (mRS) over a 2-year follow-up period. And conduct a retrospective analysis based on literature to summarize the clinical presentation, treatment selection, prognosis, and other characteristics of pediatric intracranial aneurysms.

**Results:**

All four pediatric patients successfully underwent either endovascular embolization or microsurgical clipping. Three patients achieved neurological improvement to varying degrees, while one patient with severe preoperative brain herniation died postoperatively. A total of 23 cases of pediatric intracranial aneurysms were retrieved from 21 English-language reports. Including the 4 cases in this study, the total number was 27. The male-to-female ratio was 16:11 (16 males and 11 females), and the age at onset ranged from 0.03 to 15 years (mean ± standard deviation: 6.5 ± 4.8 years). Clinical manifestations included sudden severe headache, nausea, vomiting, impaired consciousness, and so on. Initial imaging examinations showed intracranial hematoma on cranial CT (indicating ruptured intracranial aneurysms) or incidental findings on CTA/MRI (for unruptured intracranial aneurysms). The diagnosis of intracranial aneurysm was subsequently confirmed by cerebral angiography (DSA) or direct intraoperative exploration (because some patients were critically ill and could not undergo DSA). Among these, 17 were ruptured intracranial aneurysms and 6 were unruptured. Endovascular embolization was performed in 11 cases, microsurgical clipping in 10 cases, combined surgery in 1 case, and conservative treatment in 1 case. All cases in our center were ruptured intracranial aneurysms: 2 underwent endovascular treatment and 2 underwent microsurgical clipping.

**Conclusion:**

Symptoms of ruptured intracranial aneurysms in children are easily misinterpreted, necessitating precise diagnosis through multimodal imaging. Treatment strategies require individualization: endovascular therapy may be prioritized for posterior circulation or hemodynamically complex aneurysms, while surgical one-stop procedures are indicated for cases with massive hematoma or brain herniation. Long-term standardized follow-up is critical for monitoring aneurysm recurrence in pediatric patients.

## 1 Introduction

Pediatric intracranial aneurysms are rare, representing only 0.8%–5% of all intracranial aneurysms—significantly lower than in adults (1, 2). However, their clinical impact is substantial, as rupture rates in children can reach 57.6%–60.6%, leading to high mortality and disability. Posterior-circulation aneurysms and dissecting lesions account for a disproportionately higher proportion of pediatric cases compared with adults. Because the pediatric nervous system and cerebral vasculature are still in development, aneurysm rupture may result in persistent cognitive or motor deficits that influence long-term growth and quality of life (3–6). From a pathophysiological standpoint, immaturity of vascular elastic fibers and reduced tolerance to hemodynamic stress may contribute to the rapid progression of pediatric aneurysms (7). Early symptoms—such as headache, vomiting, or altered consciousness—tend to be nonspecific and are frequently misdiagnosed as common pediatric illnesses (e.g., viral encephalitis), causing delays in diagnosis. Additionally, due to the rarity of pediatric cases, existing studies are predominantly single-center and small-sample, and the lack of large-scale, long-term follow-up data leaves the natural history and risk-stratification criteria unclear, further increasing clinical uncertainty.

Endovascular embolization and microsurgical clipping for pediatric intracranial aneurysms also involve unique technical limitations. Most endovascular devices are designed for adult vessel calibers, whereas pediatric intracranial arteries are markedly smaller and more fragile, resulting in potential device–vessel mismatch and increased procedural risk. During microsurgical treatment, the immaturity and softness of pediatric brain tissue increase the likelihood of traction-related injury to the cortex and perforating arteries compared with adults. Consequently, pediatric intracranial aneurysms continue to face major unresolved issues, including the lack of early diagnostic indicators, uncertainty in selecting optimal treatment modalities, and the absence of standardized management guidelines. In this context, we retrospectively reviewed four cases of ruptured pediatric intracranial aneurysms, summarizing their clinical features, imaging characteristics, treatment strategies, and outcomes to provide guidance for individualized management.

## 2 Study population and treatment approach

### 2.1 Study population

We retrospectively reviewed pediatric patients with intracranial aneurysms who were treated at our institution between 2021 and 2025. The inclusion criteria were: (1) age ≤ 14 years; (2) a diagnosis of intracranial aneurysm confirmed by digital subtraction angiography (DSA) or intraoperative findings; and (3) the aneurysm identified as the causal lesion associated with subarachnoid hemorrhage (SAH) and/or intracerebral hemorrhage (ICH). Exclusion criteria included traumatic, infectious, or neoplastic aneurysms, as well as incomplete clinical data. A total of four patients met the criteria for analysis. All four children presented with acute neurological symptoms, most commonly severe headache and impaired consciousness, and two also exhibited nausea and vomiting. The study was authorized by the Ethics Committee of Affiliated Hospital of Jining Medical University (protocol number 2022C210), and informed approval was abandoned due to the retrospective study design.

### 2.2 Treatment methods

(1). Endovascular embolization: All procedures were performed under general anesthesia via femoral artery access using the Seldinger technique. A guiding catheter was positioned in the proximal parent artery under DSA guidance, and a microcatheter was navigated into the aneurysm using real-time roadmap assistance while minimizing contact with the aneurysm wall. Coil embolization was performed with coil size and configuration selected according to aneurysm morphology until satisfactory packing density was achieved on DSA. Balloon-assisted, dual-microcatheter, or stent-assisted techniques were applied when necessary to optimize coil stability and parent artery preservation.
(2). Microsurgical clipping: Microsurgical clipping was performed under general anesthesia through standard craniotomy approaches selected according to aneurysm location, such as the pterional or interhemispheric route. After dural opening, microsurgical dissection was used to expose the aneurysm neck and adjacent parent vessels, followed by clip placement with preservation of critical perforating arteries. In cases complicated by large intracranial hematomas or signs of herniation, hematoma evacuation and/or decompressive craniectomy was performed during the same procedure to rapidly reduce intracranial pressure(ICP) and achieve definitive hemostasis.
(3). Follow-up and monitoring: All patients were enrolled in a standardized postoperative follow-up program. Follow-up included neurological functional assessment and imaging examinations, with a focus on evaluating aneurysm occlusion status, patency of the parent artery, and the presence of residual or recurrent aneurysms. The first imaging follow-up was generally performed 3–6 months after treatment, followed by repeat examinations every 1–2 years according to risk stratification. The maximum follow-up duration was 2 years.

## 3 Results

### 3.1 Surgical outcomes

All four pediatric patients successfully underwent either endovascular embolization or microsurgical clipping. Case 1: A giant vertebral artery dissecting aneurysm was confirmed by magnetic resonance imaging (MRI) and DSA. The patient first underwent bilateral ventriculostomy, followed by parent artery occlusion and aneurysm embolization after stabilization of vital signs. Postoperatively, cerebral edema and hydrocephalus developed, necessitating posterior fossa decompression, repeat ventriculostomy, and subsequent ventriculoperitoneal shunt placement. Case 2: An anterior communicating artery (AComA) aneurysm was identified on CT and MRA, and balloon-assisted coil embolization was performed. Intraoperative angiography showed dense aneurysm occlusion with preservation of the parent artery. Case 3: A middle cerebral artery (MCA) aneurysm with associated hematoma was confirmed by CT and intraoperative findings. Microsurgical aneurysm clipping, hematoma evacuation, and decompressive craniectomy were performed. The aneurysm wall was noted to be extremely thin-walled, but successful clipping was achieved. Case 4: MRI performed two years earlier had indicated an AComA aneurysm. Following rupture, the patient presented with bilateral fixed dilated pupils and deep coma. Emergent hematoma evacuation, aneurysm clipping, and decompressive craniectomy were performed; however, the patient remained comatose postoperatively with absent light reflexes. The family elected to discontinue further resuscitative efforts.

### 3.2 Prognosis and follow-up findings

At discharge, the patients demonstrated varying degrees of neurological recovery. Case 1 had a discharge mRS score of 4. At the 2-year follow-up, right-sided motor function improved to Brunnstrom Stage IV. Case 2 was discharged with an mRS score of 1. Follow-up DSA at 8 months revealed recurrence at the aneurysm neck, and retreatment with a WEB device was planned. Case 3 had a discharge mRS score of 3. After one year of rehabilitation, the patient showed marked improvement in both language and limb function and subsequently underwent successful cranioplasty. Case 4 developed postoperative brain herniation and died despite aggressive resuscitation Table 1.

**Table 1:** Baseline characteristics, imaging findings, treatment strategies, and outcomes of the four pediatric patients with intracranial aneurysms.

Items	Case 1	Case 2	Case 3	Case 4
Age(years)	8	10	13	10
Sex	Male	Female	Male	Male
Presenting symptoms	Headache, fever, convulsions, loss of consciousness	Sudden loss of consciousness, head trauma	Sudden headache, impaired consciousness	Sudden headache, convulsions, loss of consciousness
GCS score on admission	5 (E1V1M3)	11 (E4V1M6)	5 (E1V1M3)	6 (E1V1M4)
Hunt-Hess grade	IV	III	IV	V
Location	vertebral artery (V4 segment)	AComA	Left MCA bifurcation	Left AComA
Aneurysm morphology	Dissecting aneurysm	Saccular aneurysm with daughter sac	Saccular aneurysm with pseudoaneurysm formation	Saccular aneurysm with pseudoaneurysm formation
Maximum aneurysm Size(mm)	41.2 × 14.6 × 11.6	4.2 × 2.9 × 1.7	7 × 6 × 6	5 × 5 × 4
Ruptured	Yes (SAH with IVH)	Yes (SAH)	Yes (SAH with ICH)	Yes (SAH)
Initial imaging modalities	MRI, TCD (Figures 1A,B)	CT, MRI (Figures 9, 10)	CT (Figure 14)	MRI, CT (Figures 18–20)
Definitive diagnostic modality	CTA, DSA (Figures 2, 3)	DSA (Figures 11A,B)	Craniotomy exploration	Craniotomy exploration
Treatment modality	Endovascular Treatment	Endovascular Treatment	Microsurgical clipping	Microsurgical clipping
Procedural details	Occlusion of the parent artery and aneurysm embolization, decompressive craniectomy, ventriculostomy, ventriculoperitoneal shunt (Figures 4–7)	Balloon-assisted coil embolization (Figure 12)	Aneurysm Clipping + Hematoma Evacuation + Decompressive Craniectomy (Figures 15, 16)	Intracranial hematoma evacuation + left AComA aneurysm clipping + decompressive craniectomy
mRS score at discharge	4	1	3	–
Postoperative complications	Hydrocephalus, Pneumonia	None	None	–
Follow-up outcomes	No recurrence on 2-year DSA; well-established collateral circulation (Figures 8A–C)	Aneurysm neck recurrence on 8-month DSA (Figures 13A–D)	Normal vessel course on CTA; cranioplasty at 1 year (Figure 17)	Death
Long-term functional outcome	Right upper limb Brunnstrom stage IV at 2 years	No neurological sequelae	Marked improvement in language and limb function at 1 year	–

**Figure 1:**
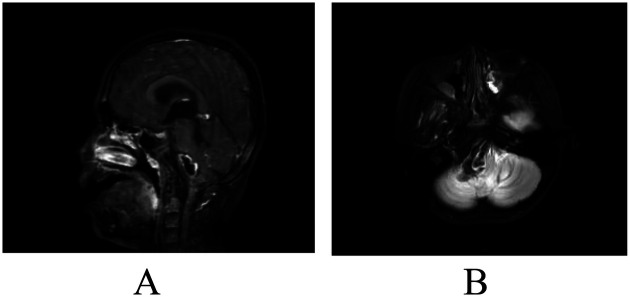
**(A,B)** MRI demonstrates a fusiform dilatation of the right vertebral artery (V4 segment) measuring approximately 14 × 14 × 31 mm, with compression and leftward displacement of the adjacent medulla and pons.

**Figure 2:**
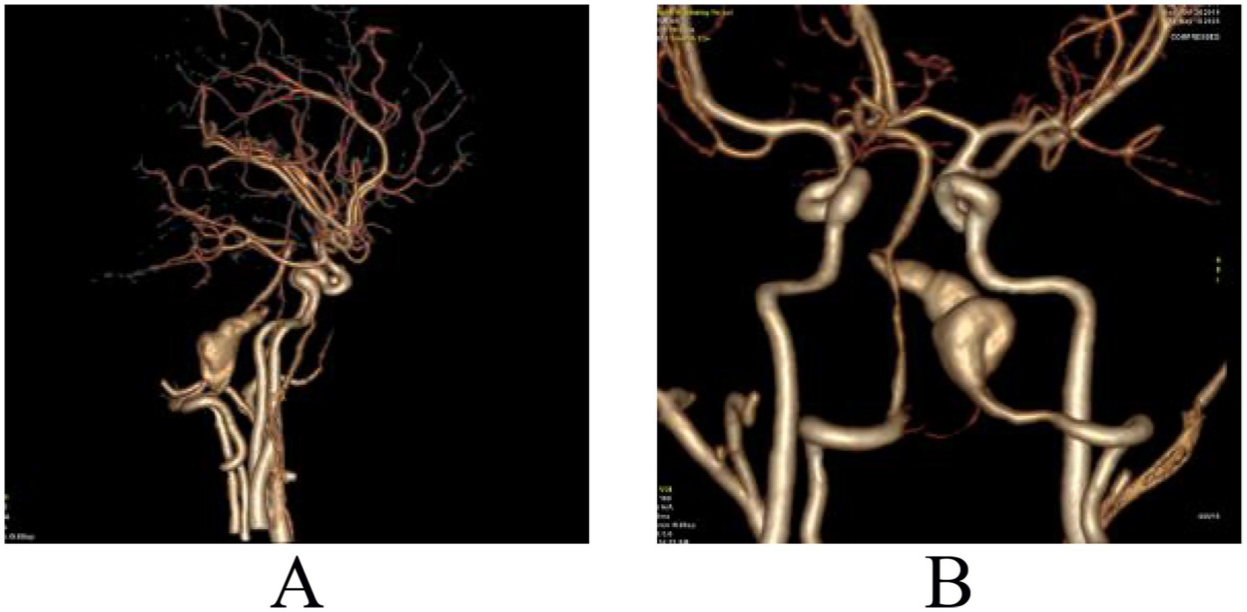
**(A,B)** 3D CTA reconstruction reveals irregular dilatation and wall thickening of the right vertebral artery (V4 segment), extending approximately 35 mm in length.

**Figure 3:**
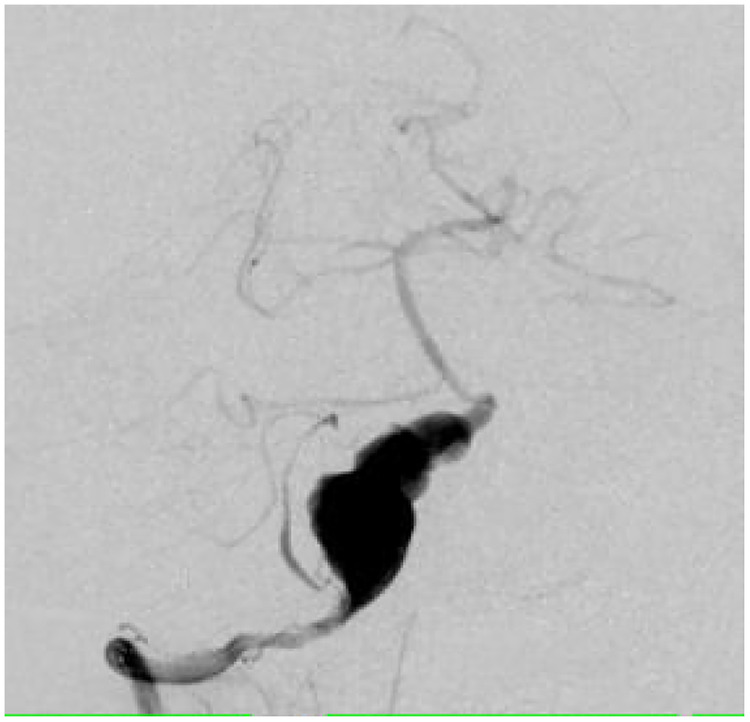
DSA confirms a giant dissecting aneurysm of the right vertebral artery (V4 segment), measuring 41.2 × 14.6 × 11.6 mm, with irregular morphology.

**Figure 4:**
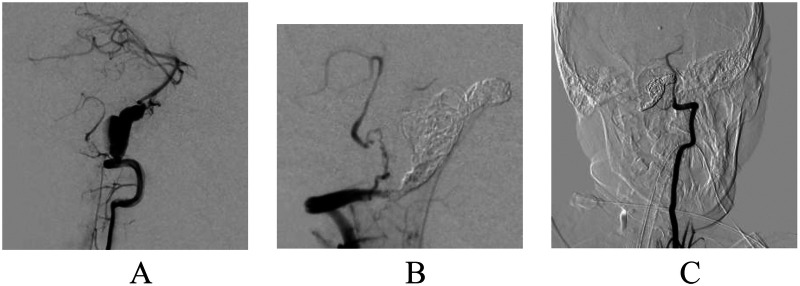
**(A–C)** Post-embolization DSA demonstrates complete occlusion of the aneurysm and parent artery.

**Figure 5:**
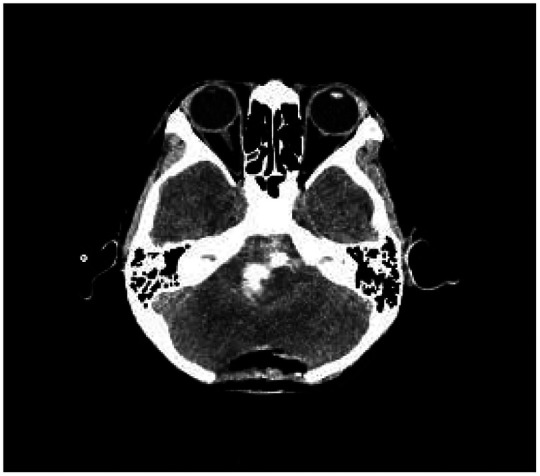
Postoperative CT shows hyperdense embolization material within the posterior fossa following endovascular treatment.

**Figure 6:**
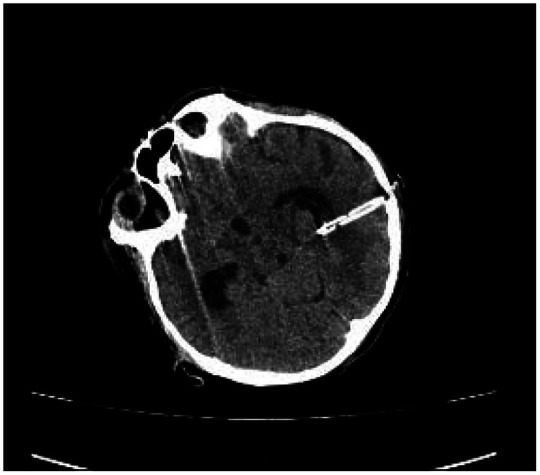
CT reveals an external ventricular drainage catheter positioned in the posterior horn of the left lateral ventricle.

**Figure 7:**
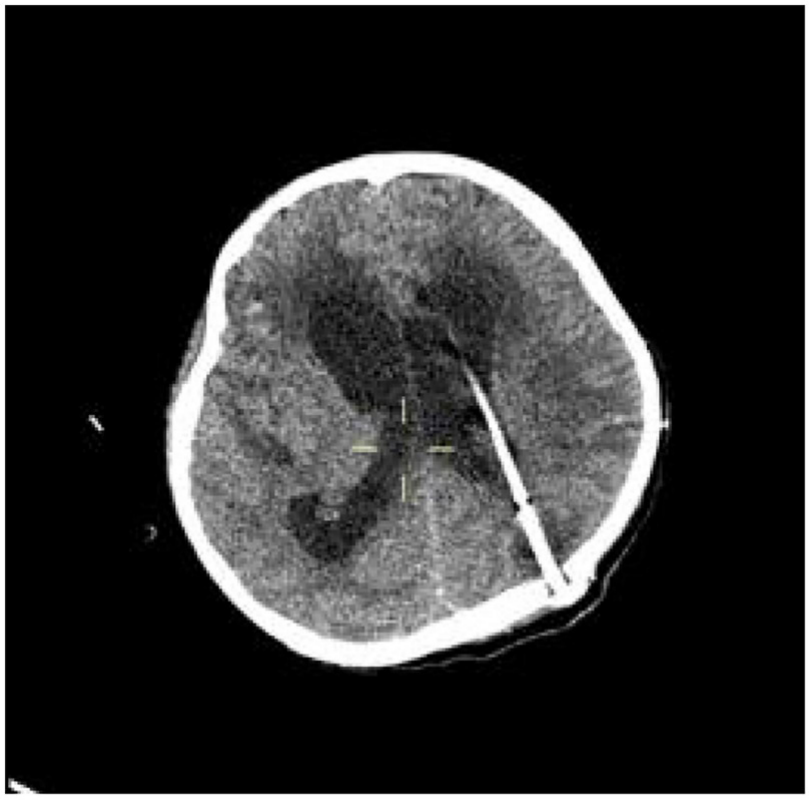
CT demonstrates a left parietal burr hole with the ventricular catheter extending into the anterior horn of the left lateral ventricle.

**Figure 8:**
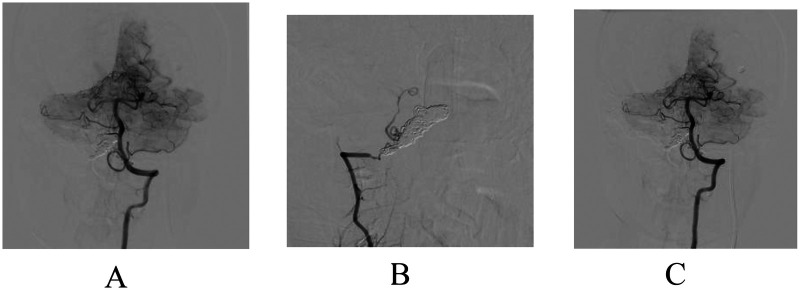
**(A–C)** Follow-up DSA at 2 years shows no visualization of the aneurysm or distal right vertebral artery, with collateral perfusion supplied via the left vertebral artery.

**Figure 9:**
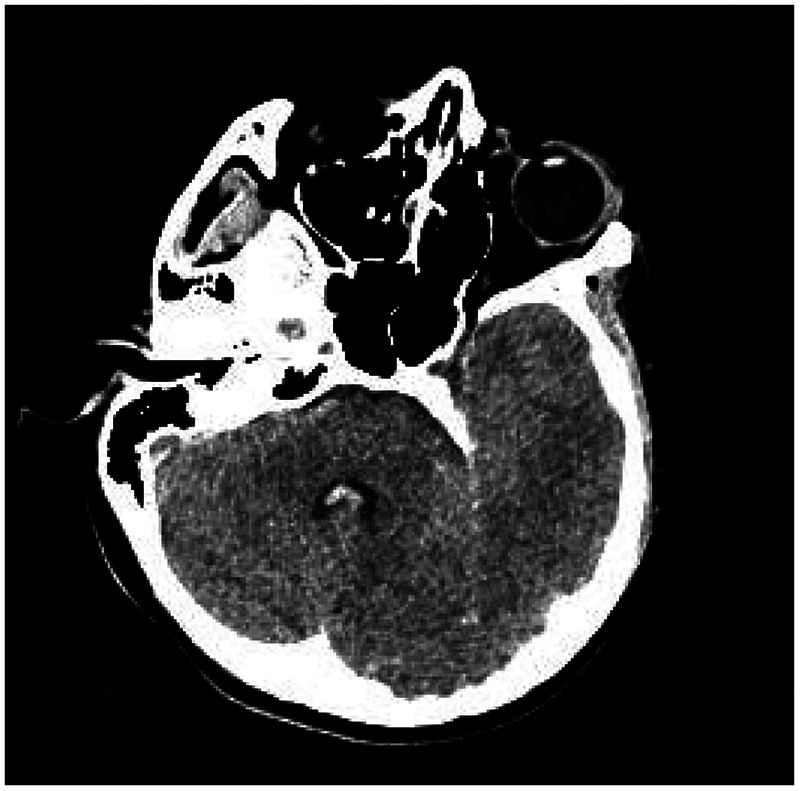
CT shows subarachnoid hemorrhage predominantly within the basal cisterns, with minimal intraventricular hemorrhage.

**Figure 10:**
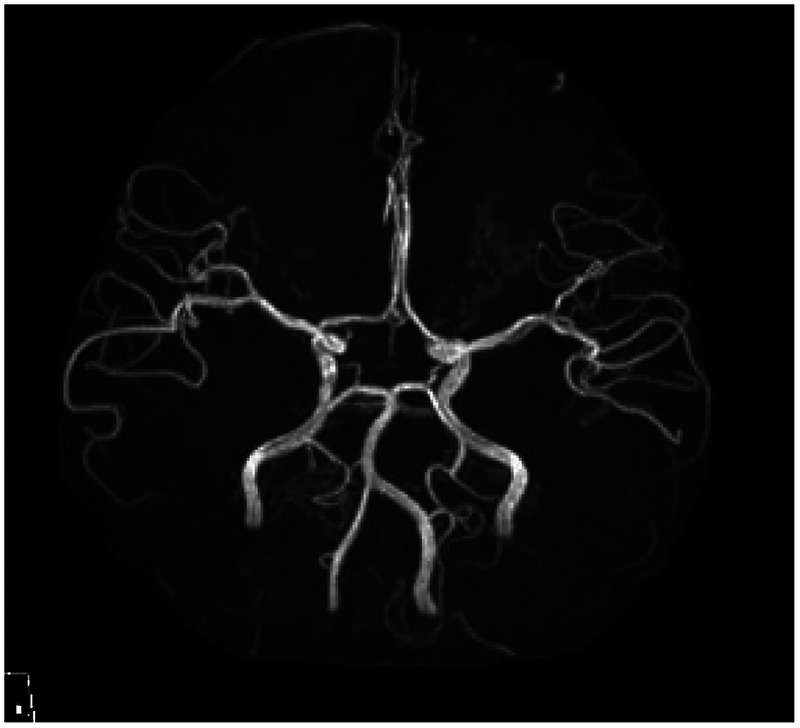
MRA reveals a saccular aneurysm arising from the AComA, measuring approximately 3.0 mm in diameter.

**Figure 11:**
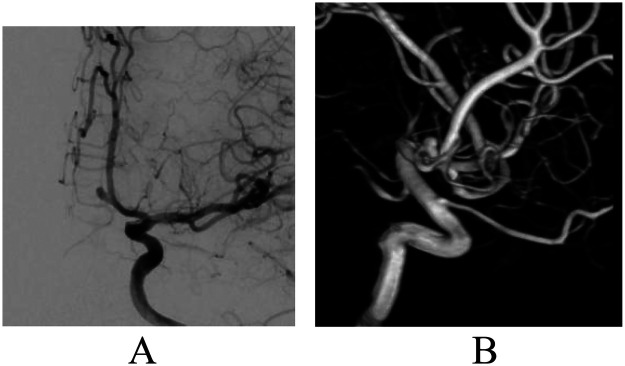
**(A,B)** DSA demonstrates a saccular aneurysm of the AComA measuring 4.2 × 2.9 × 1.7 mm with an irregular neck.

**Figure 12:**
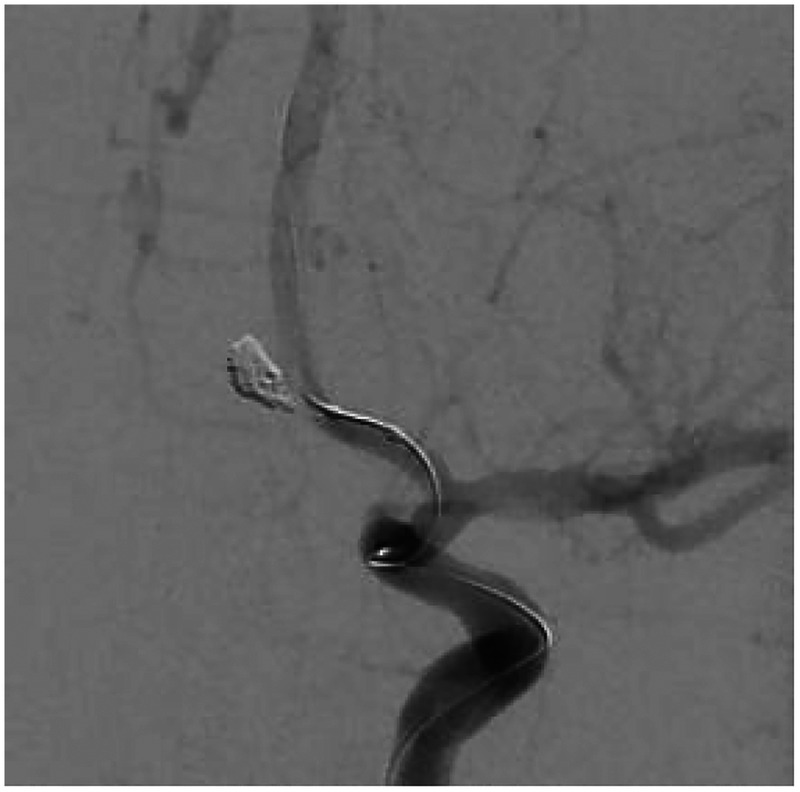
Post–balloon-assisted coil embolization angiography demonstrates dense aneurysm occlusion with preservation of the parent artery.

**Figure 13:**
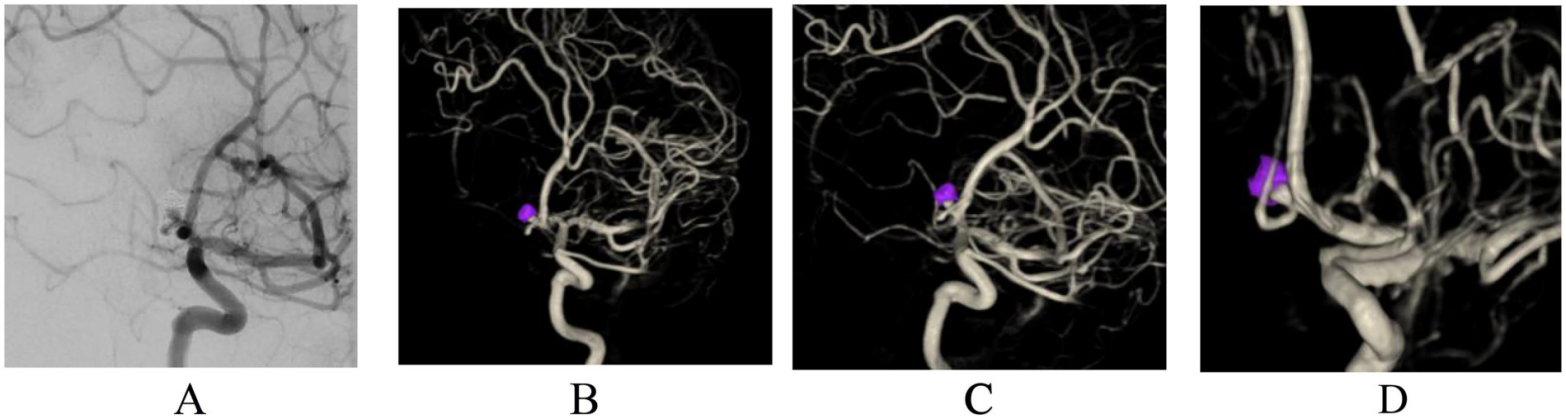
**(A–D)** Follow-up DSA at 8 months shows residual filling at the aneurysm neck, consistent with aneurysm recurrence.

**Figure 14:**
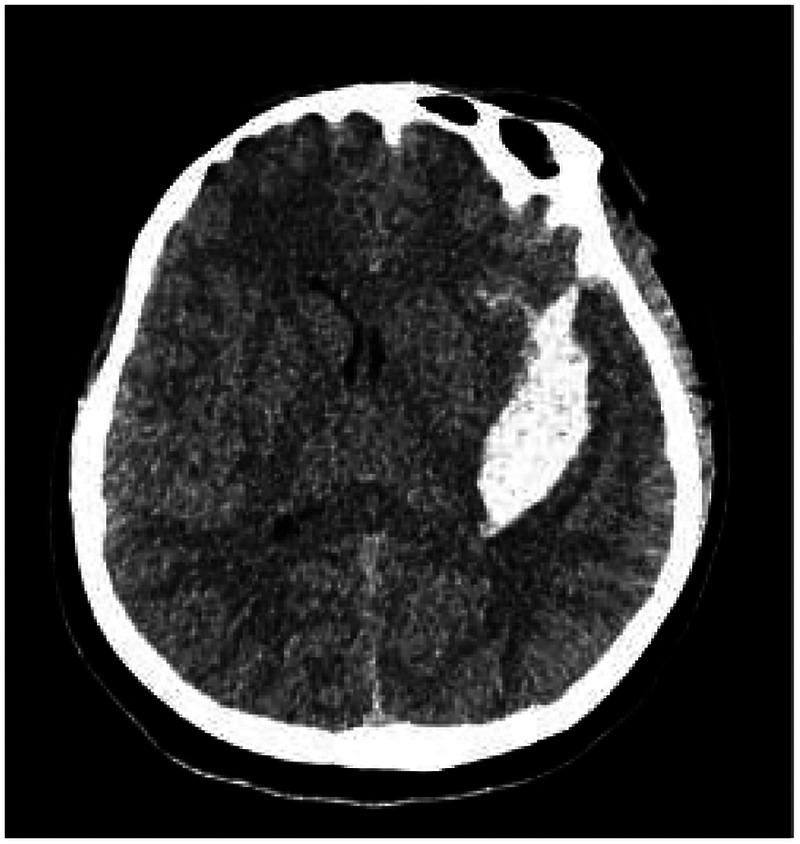
CT demonstrates a hyperdense lesion in the left temporal-insular region with surrounding edema, consistent with intracerebral hemorrhage causing mild midline shift.

**Figure 15:**
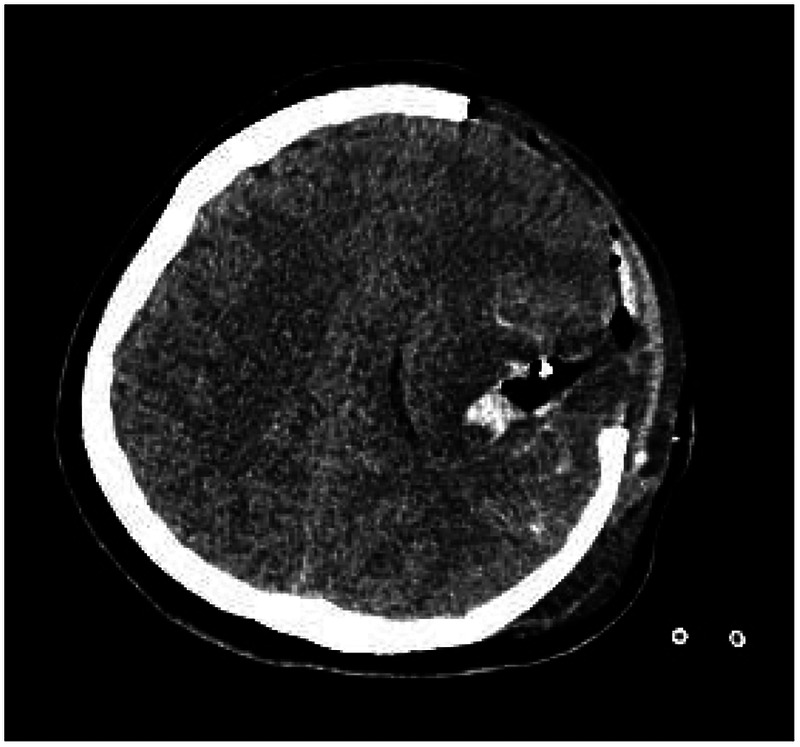
Postoperative CT shows a left frontotemporal craniectomy with intracranial air, surgical clips, and drainage catheters *in situ*.

**Figure 16:**
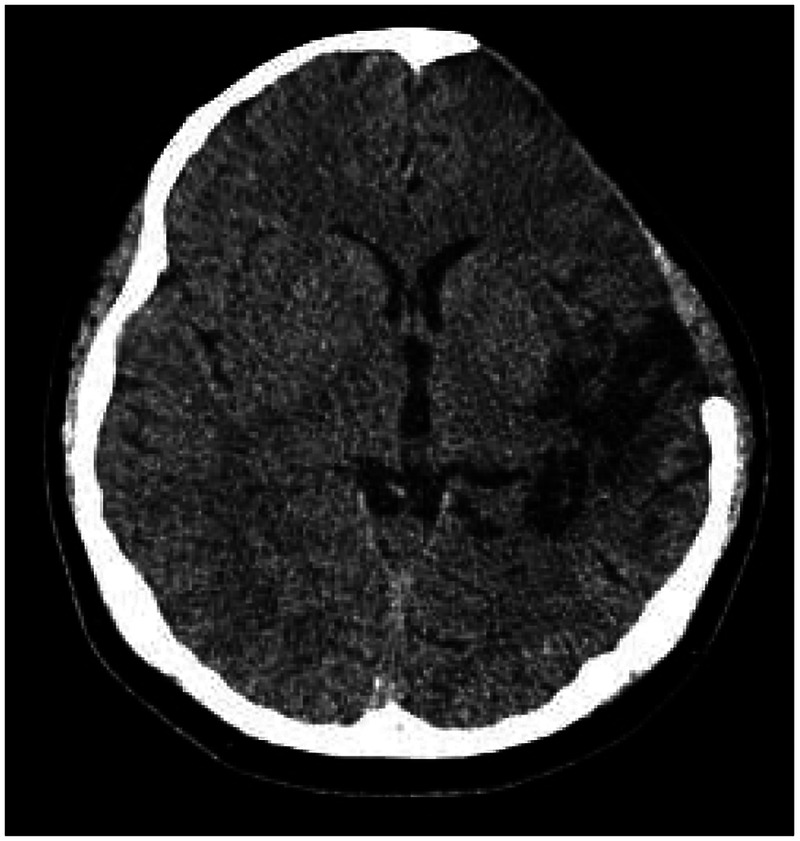
CT obtained 2 months postoperatively shows resolution of mass effect without evidence of cerebral herniation.

**Figure 17:**
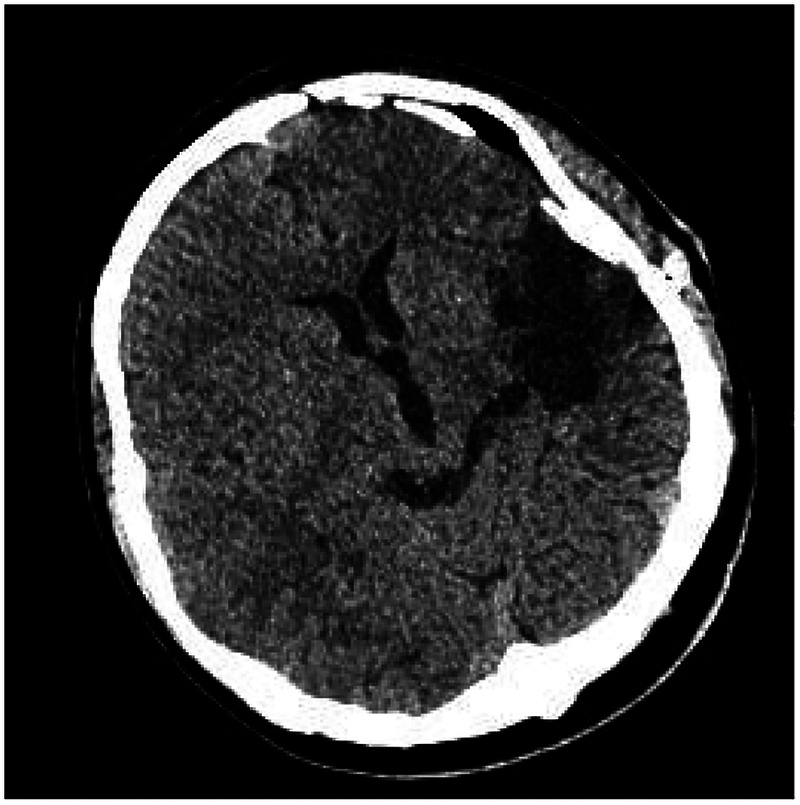
CT after cranioplasty demonstrates restoration of the cranial defect.

**Figure 18:**
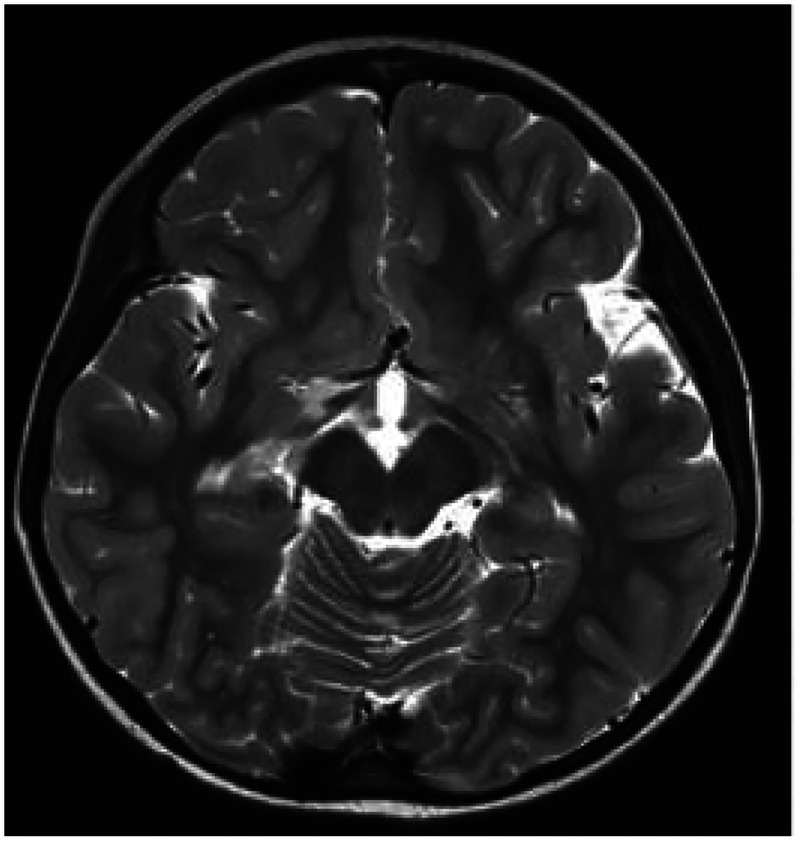
Pre-rupture MRI demonstrates an AComA aneurysm with associated congenital midline anomalies.

**Figure 19:**
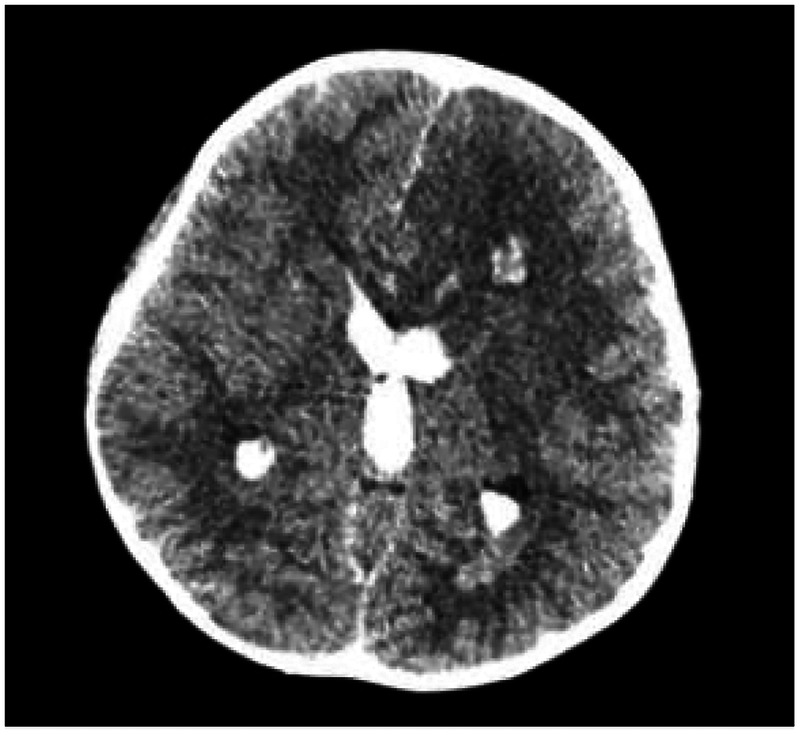
CT shows extensive intracerebral hemorrhage with marked midline shift of transtentorial herniation.

**Figure 20:**
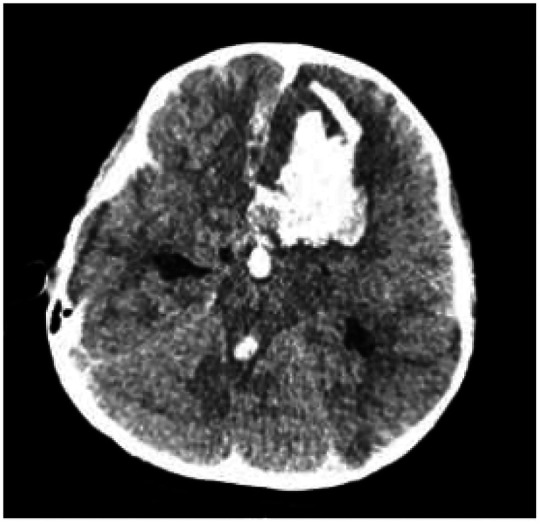
CT shows extensive intraventricular hemorrhage with marked signs of transtentorial herniation.

## 4 Literature review

We searched the PubMed database for similar pediatric intracranial aneurysm case reports published between January 1, 2021 and December 31, 2025. The keywords used are as follows: “intracranial aneurysm in children”, “endovascular treatment”, “microsurgical clipping”, “ruptured intracranial aneurysm”, “unruptured intracranial aneurysm”. Collect all case reports related to intracranial aneurysms in children. Based on literature search, summarize the social demographics (gender, age), clinical manifestations (headache, dizziness, nausea and vomiting, consciousness disorders, other manifestations), imaging examinations, follow-up, and prognosis of the relevant cases.

### 4.1 Results

A total of 21 English literature reports were retrieved, involving 23 cases of pediatric intracranial aneurysms. The ratio of male to female patients was 13:10 (13 males and 10 females), and the age at onset ranged from 0.03 to 15 (6.5 ± 4.8) years. Clinical manifestations included sudden severe headache, nausea, vomiting, and consciousness disorders. Initial imaging examinations: Cranial CT indicated intracranial hematoma (ruptured intracranial aneurysm), CTA/MRI examination (unruptured intracranial aneurysm), followed by cerebral angiography (DSA) or direct craniotomy exploration surgery (due to the critical condition of some patients, DSA could not be performed). Among them, 17 cases were ruptured intracranial aneurysms and 6 cases were unruptured intracranial aneurysms. 11 cases underwent endovascular embolization, 10 cases underwent microsurgical aneurysm clipping surgery, 1 case received combined surgery treatment, and 1 case received conservative treatment. 17 cases had a good prognosis, 5 cases had mild neurological dysfunction, and 1 case died.

## 5 Discussion

This study analyzed four pediatric intracranial aneurysm cases and highlighted several distinctive clinical characteristics, including nonspecific initial manifestations, rapid clinical deterioration after rupture, and a high susceptibility to misdiagnosis, such as meningitis or traumatic brain injury. These features appear to be closely associated with the high prevalence of posterior circulation and dissecting aneurysms in children, as well as the structural and physiological immaturity of the pediatric vascular wall. Collectively, these factors contribute to the unique clinical course and management challenges of pediatric intracranial aneurysms.

### 5.1 Clinical and imaging characteristics of pediatric intracranial aneurysms

#### 5.1.1 Differences between pediatric and adult intracranial aneurysms

The clinical progression of pediatric intracranial aneurysms differs fundamentally from that observed in adults. In this cohort, one patient was initially misdiagnosed with meningitis because of headache, fever, and bloody cerebrospinal fluid (CSF), illustrating how nonspecific symptoms can easily be mistaken for infectious diseases. The patient's giant dissecting aneurysm in the right vertebral artery (V4 segment) progressed to rupture within five days, suggesting an aggressive disease course. This rapid deterioration may be attributed to the strong hemodynamic stress of posterior circulation blood flow combined with the limited tensile strength of the immature pediatric vascular wall (8). Similarly, Case 3 demonstrated an abrupt clinical decline, progressing from headache to brain herniation within six hours. This course highlights the extreme danger of rapidly elevated ICP when aneurysmal rupture is complicated by intracerebral hematoma formation. The presence of a pronounced Cushing response, including markedly elevated blood pressure (180/90 mmHg) on admission and a Cushing index of 3.21, is associated with an exceptionally high in-hospital mortality risk and a high likelihood of requiring emergency craniotomy (9).

Epidemiological data indicate that posterior circulation aneurysms are significantly more prevalent in pediatric patients than in adults, with a notably higher proportion of dissecting aneurysms, particularly involving the vertebral artery (1, 3, 8, 10–12). This distribution differs markedly from adult intracranial aneurysms, which are predominantly associated with chronic vascular injury processes such as atherosclerosis and typically follow a prolonged degenerative course. In contrast, pediatric intracranial aneurysms are more often related to idiopathic or congenital vascular wall abnormalities. Immaturity of the arterial media, characterized by underdeveloped elastic fibers and reduced tolerance to hemodynamic stress, may predispose pediatric vessels to structural instability. Disruption of elastin–collagen cross-linking, accelerated elastin degradation, and extracellular matrix imbalance may collectively weaken the arterial wall, rendering it more susceptible to shear stress and dissection. These intrinsic vulnerabilities form the pathological basis for the high susceptibility of pediatric intracranial aneurysms—particularly dissecting lesions—to rapid progression under posterior circulation hemodynamic forces (11, 13). Case 4 in our cohort further supports this concept. The patient had a previously identified AComA aneurysm on MRI but did not receive timely treatment. Following rupture, bilateral pupillary dilation and loss of consciousness developed rapidly, indicating abrupt ICP escalation and irreversible brain herniation. This case illustrates that even small-to-medium asymptomatic aneurysms in children may rupture suddenly under hemodynamic fluctuations. Notably, none of the pediatric patients in this cohort had a history of significant trauma, supporting congenital structural vulnerability or hemodynamic abnormalities as primary etiological factors. Because early clinical manifestations are often nonspecific, misdiagnosis is common, underscoring the necessity for careful imaging-based differentiation between primary vascular lesions and secondary neurological conditions (14).

#### 5.1.2 Imaging features

The morphological presentation of pediatric intracranial aneurysms is more complex and heterogeneous than that observed in adults. Our cases demonstrate that pediatric intracranial aneurysms show significant morphological heterogeneity. Such morphological complexity underscores the clinical necessity of high-resolution vessel wall imaging (HR-VWI), which enables accurate identification of subtle pathological features, including dissection planes and intramural hematomas. By overcoming the spatial resolution limitations of conventional imaging modalities, HR-VWI plays a critical role in reducing missed or delayed diagnoses in pediatric patients (15, 16). In this study, a multimodal imaging strategy was adopted, incorporating computed tomography angiography (CTA) for rapid aneurysm screening, MRI for evaluation of hematomas and hydrocephalus, and DSA for detailed visualization of aneurysm morphology and parent vessel anatomy. This integrated approach provides robust diagnostic support for individualized treatment planning and is consistent with the concept of stepwise imaging evaluation in cerebrovascular disease management (17).

### 5.2 Treatment strategy selection

#### 5.2.1 Posterior circulation aneurysms

Posterior circulation aneurysms, including lesions involving the vertebral and basilar artery systems, pose substantial therapeutic challenges in pediatric patients because of their close proximity to the brainstem, cerebellum, and critical perforating vessels. Using the vertebral artery V4 segment dissecting aneurysm in Case 1 as an example, several anatomical factors contributed to the high surgical risk. First, the aneurysm was located adjacent to the dorsal medulla, necessitating cerebellar retraction during microsurgical exposure, which substantially increases the risk of injury to the posterior inferior cerebellar artery (PICA) and lower cranial nerves, potentially resulting in severe complications such as dysphagia and respiratory dysfunction (18). Second, the aneurysm was massive (41.2 mm × 14.6 mm × 11.6 mm) and exhibited fusiform dilation, rendering complete exclusion by conventional clip application technically challenging and increasing the risk of intraoperative rupture due to the fragile arterial wall. Third, pediatric posterior circulation vessels are characteristically slender, with vertebral artery diameters often measuring <3 mm, which further elevates the risk of vascular injury during craniotomy exposure compared with adult patients. Given these anatomical constraints, endovascular embolization has emerged as the preferred therapeutic option for complex posterior circulation aneurysms in pediatric patients (19). In Case 1, dual-microcatheter super-selective embolization with parent artery occlusion was performed, effectively excluding the aneurysm from circulation while avoiding direct brainstem manipulation. This strategy relies on coil-induced mechanical flow diversion and preservation of distal perfusion through collateral circulation. Accumulating evidence indicates that, for pediatric vertebral artery dissecting aneurysms, endovascular treatment achieves increasing technical success rates and is associated with a lower incidence of postoperative neurological deficits compared with microsurgical approaches, further supporting this treatment rationale (20).

#### 5.2.2 Anterior circulation aneurysms

Anterior circulation aneurysms, such as those involving the AComA or anterior cerebral artery, generally allow greater flexibility in treatment selection because of their relatively superficial location and less complex surrounding anatomy compared with posterior circulation lesions. Nevertheless, therapeutic decision-making must comprehensively consider aneurysm size, morphology, and hemodynamic characteristics. The AComA aneurysm in Case 2 (4.2 mm × 2.9 mm × 1.7 mm) exhibited several anatomical and clinical features associated with lower procedural risk. First, the lesion was located anterior to the circle of Willis and distant from critical structures such as the brainstem, allowing safe exposure through standard transfrontal or pterional approaches. In addition, endovascular access through the internal carotid artery was straightforward. Second, the absence of a significant intracranial hematoma eliminated the need for urgent decompressive treatment. Third, despite its irregular morphology and the presence of a subsidiary sac, the aneurysm did not involve critical perforating arteries. For small-to-medium anterior circulation aneurysms with such characteristics, endovascular treatment offers distinct advantages (21). In this case, balloon-assisted coil embolization was performed with pediatric-specific technical considerations. Because the AComA in children is often smaller in caliber than in adults, appropriately sized microcoils (1.5 mm × 3 cm) were selected to minimize mechanical irritation of the vascular wall and reduce the risk of postoperative vasospasm. Temporary balloon-assisted remodeling of the parent artery helped prevent coil protrusion while improving embolization density. Compared with microsurgical clipping, this endovascular strategy avoids frontal lobe traction and is therefore more consistent with the goal of preserving cognitive and neurodevelopmental function in pediatric patients.

#### 5.2.3 Aneurysms with acute complications

When aneurysm rupture is accompanied by acute complications such as large intracranial hematoma or impending brain herniation, the urgency of the clinical condition becomes the dominant determinant of treatment selection, often outweighing considerations related to aneurysm location or vascular access (21). In Cases 3 and 4, involving MCA and AComA aneurysms, patients presented with typical acute manifestations. Under such circumstances, the primary therapeutic goals are rapid intracranial decompression and definitive hemostasis. Although aneurysms of the MCA and AComA are located within the anterior circulation and may be amenable to endovascular treatment under stable conditions (22), the presence of a massive intracranial hematoma often shifts the treatment strategy toward microsurgical clipping. The indispensable role of craniotomy in this setting is reflected in three key aspects. First, decompressive craniectomy allows immediate reduction of ICP, interrupting the vicious cycle of hematoma expansion, cerebral edema, and secondary ischemic injury. Second, direct surgical visualization permits complete evacuation of the hematoma and elimination of mass effect, which cannot be achieved by endovascular techniques alone. Third, microsurgical exposure enables precise dissection of interhemispheric or sylvian fissure vessels and secure clipping of bifurcation aneurysms while preserving critical perforating arteries, such as the lenticulostriate arteries (21). This integrated “one-stop” surgical strategy is particularly critical in pediatric emergencies, as sustained intracranial hypertension may result in irreversible neuronal injury despite the relatively strong compensatory capacity of the developing brain.

#### 5.2.4 Individualized trade-offs for special anatomical locations

Beyond typical aneurysm locations, pediatric intracranial aneurysms may arise in less common sites, such as the distal posterior cerebral artery or anterior choroidal artery, necessitating more nuanced and individualized treatment strategies. For small, unruptured aneurysms adjacent to the basal ganglia, endovascular embolization with super-selective microcatheterization may reduce interference with critical perforating vessels. In contrast, when an aneurysm exhibits a saccular protrusion and is located near eloquent motor pathways, microsurgical clipping may allow more precise manipulation and better preservation of surrounding neural fiber tracts. The relative immaturity of elastic fibers in pediatric vessel walls further accentuates the importance of aneurysm location in treatment planning. Posterior circulation arteries typically have thinner walls and a higher density of perforating branches, making them particularly susceptible to vasospasm or rupture during surgical traction. Conversely, although anterior circulation vessels are more superficially located, their smaller calibers increase the technical difficulty of endovascular device navigation. These anatomical and developmental characteristics collectively underscore the need for a balanced, location-specific approach to treatment selection. Accordingly, preservation of normal vascular structures should be regarded as a central principle in surgical planning for pediatric intracranial aneurysms. Even in emergency settings, undue injury to intact vessels should be minimized rather than sacrificed solely for rapid hemostasis, as long-term neurological outcomes in children depend heavily on meticulous vascular protection (23). For this purpose, we searched case reports on pediatric intracranial aneurysms published in the past five years and summarized the relevant treatment strategies as follows (Table 2).

**Table 2:** Literature review of pediatric intracranial aneurysms.

First author	Country	Gender	Age (year)	Aneurysm location	Ruptured	Treatment	Outcome
Rashmi Saraf et al. (24)	IND	F	11	L-MCA	Y	Endovascular	Good neurological recovery
Masja Bluhme Hoe et al. (25)	DNK	F	10	L-PCA	Y	Microsurgery	Mild cognitive impairment
Chao Peng et al. (26)	CHN	F	4	R-MCA	N	Microsurgery	No neurological deficit
Akram M Eraky et al. (27)	USA	F	15	L-ICA	N	Endovascular	Aneurysm nearly occluded
Akram M Eraky et al. (27)	USA	M	15	L-ICA	N	Endovascular	Aneurysm completely occluded
Surya Kant et al. (28)	IND	M	10	BA	Y	Endovascular	Good collateral circulation
Ehsan Hosseini et al. (29)	IRN	F	4	L-ICA	N	Endovascular	Uncomplicated recovery
Vinayagamani et al. (30)	IND	M	9	ACoA	Y	Endovascular	Normal neurodevelopment
Fatemeh Safari et al. (31)	IRN	M	10	L-ICA	Y	Endovascular	Symptoms resolved
Aghamiri et al. (19)	IRN	F	10	L-PCA	Y	Endovascular	Uncomplicated recovery
Zeferino et al. (32)	BRA	F	2	R-ICA	Y	Endovascular	No epilepsy, recovered well
Cristina et al. (33)	ROU	M	4	ACoA	Y	Microsurgery	Normal development
Hayato et al. (22)	JPN	M	3	R-MCA	Y	Endovascular	No obvious deficit
Livshits et al. (34)	RUS	M	5	L-MCA	N	Microsurgery	Normal neurological function
Livshits et al. (34)	RUS	F	2	L-ACA	N	Microsurgery	Uncomplicated recovery
Regina et al. (35)	PRT	F	0.2	L-MCA	Y	Microsurgery	Mild hemiparesis remained
Daniela et al. (36)	ARG	M	0.1	L-ACA	Y	Conservative	No neurological deficit
Pin Fee Chong et al. (37)	JPN	M	10	L-PCA	Y	Combined	Death
George et al. (38)	USA	M	1.3	L-ACA	Y	Microsurgery	Mild strabismus remained
Anirudh et al. (39)	IND	M	0.8	R-MCA	Y	Microsurgery	Improved motor function
Chaojue Huang et al. (40)	CHN	M	8	R-AICA	Y	Microsurgery	No sequelae
Rauf Hamid et al. (41)	TUR	F	0.03	L-ACA	Y	Endovascular	Mild hypotonia
Anurag et al. (42)	IND	M	0.2	L-MCA	Y	Microsurgery	No recurrence

### 5.3 Complication management and pediatric specificity

The incidence and clinical impact of postoperative complications in pediatric intracranial aneurysms demonstrate distinct age-related characteristics, necessitating management strategies that fully account for the physiological immaturity of the developing brain and cerebral vasculature.

Although the overall incidence of cerebral vasospasm after ruptured intracranial aneurysms is reported to be lower in children than in adults, its occurrence may be associated with a higher risk of ischemic brain injury. Experimental and clinical studies suggest that inflammatory mediators released after SAH, such as interleukin-1, may contribute to nitric oxide depletion and subsequent vasoconstriction. Immature pediatric vessel walls appear more sensitive to vasoactive stimuli, potentially amplifying ischemic damage once vasospasm develops (43). From a clinical perspective, close monitoring of consciousness level and cerebral hemodynamic status is recommended in the postoperative period. In patients with deteriorating mental status or new-onset motor deficits, prompt cerebral angiography should be considered to identify vasospasm. Early administration of calcium channel blockers such as nimodipine may improve vasodilatory function, while endovascular balloon angioplasty can be employed in refractory cases (13, 44).

Pediatric brain tissue is characterized by heightened excitability, and postoperative factors including hematoma compression, cerebral edema, and surgical manipulation may precipitate epileptic seizures (45). Failure to achieve timely seizure control can result in secondary hypoxic injury and adversely affect neurological recovery. Accordingly, prophylactic antiepileptic therapy should be individualized based on seizure type and risk profile, with careful consideration of potential long-term effects on cognitive development. Continuous or long-term electroencephalography (EEG) monitoring is recommended for high-risk patients to facilitate early detection and treatment, thereby improving overall prognosis (46).

In this cohort, three pediatric patients developed varying degrees of postoperative hydrocephalus, underscoring the clinical relevance of this complication in children with ruptured intracranial aneurysms. The presence of intraventricular hemorrhagic CSF can disrupt choroid plexus function through mechanical obstruction, oxidative injury, and inflammatory responses, thereby impairing normal CSF absorption. These mechanisms contribute to persistent communicating hydrocephalus, which may further compromise cognitive function, accelerate neurological deterioration, and adversely affect long-term prognosis in pediatric patients (47). Compared with adults, hydrocephalus in children tends to progress more rapidly and is associated with a higher risk of intracranial hypertension and brain herniation, emphasizing the importance of early treatment. External ventricular drainage provides rapid ICP reduction during the acute phase and facilitates subsequent assessment for definitive shunt placement. During ventriculoperitoneal shunting, catheter length and configuration should be tailored to cranial and abdominal growth patterns to balance immediate efficacy with long-term developmental considerations.

Children also exhibit increased susceptibility to postoperative infections because of immune system immaturity, prolonged hospitalization, invasive procedures, and frequent exposure to broad-spectrum antibiotics. Immature skin and mucosal barriers, together with indwelling devices such as intracranial shunts, endotracheal tubes, and tracheostomies, increase opportunities for pathogen colonization (48). In addition, prolonged broad-spectrum antibiotic use may disrupt normal microbial flora, predisposing pediatric patients to multidrug-resistant infections.

Accordingly, postoperative management should emphasize early detection and targeted treatment. Infection surveillance strategies include routine sputum, blood, and CSF cultures to dynamically monitor pathogen profiles; avoidance of unnecessary broad-spectrum antibiotic use; and timely adjustment of antimicrobial therapy based on susceptibility testing (49). More broadly, close monitoring of ICP for hydrocephalus, vigilant preventive care during prolonged hospitalization, and individualized adjustment of monitoring frequency and treatment strategies for complications such as vasospasm and epilepsy are essential. A comprehensive, pediatric-specific complication management approach may mitigate adverse neurological outcomes and optimize long-term functional recovery.

### 5.4 Follow-up strategy and long-term management

#### 5.4.1 Early monitoring for aneurysm recurrence

Pediatric intracranial aneurysms are associated with a relatively high risk of recurrence, particularly in dissecting aneurysms. Previous studies have reported that recurrence rates of pediatric vertebral artery dissecting aneurysms may be as high as 69%, exceeding those observed in adult populations (14). Early aneurysm recurrence is frequently asymptomatic, underscoring the importance of imaging-based follow-up strategies. A vascular imaging–centered surveillance protocol is therefore recommended, with initial DSA performed at 3–6 months post-treatment to confirm aneurysm occlusion and assess collateral circulation, followed by biennial or triennial imaging based on clinical stability. In high-risk cases, such as dissecting aneurysms or previously ruptured aneurysms—where rebleeding risk has been reported to approach 60%—shorter follow-up intervals should be considered (1, 17, 21, 22, 50). Dynamic imaging surveillance enables early identification of aneurysm recurrence or residual aneurysm enlargement, thereby preserving an optimal window for timely treatment (51). Long-term management of pediatric intracranial aneurysms should prioritize recurrence surveillance, as recurrence rates are generally higher than those observed in adults and exhibit heterogeneity across aneurysm subtypes.

Additionally, the ongoing development of pediatric vasculature may further influence recurrence patterns. As children grow, dynamic changes in cerebral hemodynamics may destabilize previously treated or quiescent aneurysms through altered shear stress. Consequently, follow-up should not be limited to the original aneurysm site but should also include screening for *de novo* aneurysm formation, particularly in patients with suspected congenital vascular wall abnormalities, in whom the entire cerebrovascular system may be at risk. Regular long-term imaging follow-up facilitates early detection of recurrent or newly formed aneurysms, allowing appropriate timing of secondary treatment and reducing the likelihood of catastrophic outcomes associated with delayed treatment, including recurrent aneurysm rupture (50).

#### 5.4.2 Timely treatment for long-term complications

Postoperative complications in pediatric intracranial aneurysm patients may present in a delayed manner or persist over the long term. Systematic follow-up therefore plays a critical role in the early detection and timely management of these complications. In children with hydrocephalus treated with ventriculoperitoneal shunt placement, long-term surveillance of shunt function is essential. With ongoing somatic growth, shunt-related complications such as catheter length insufficiency or overdrainage may occur, manifesting clinically as low-pressure symptoms (e.g., headache and vomiting) or recurrent ventricular enlargement. Regular imaging assessments combined with clinical symptom evaluation allow timely adjustment or revision of the shunt system. In addition, epileptic seizures may develop months or even years after aneurysm treatment. Follow-up protocols should therefore incorporate systematic assessment of seizure history and periodic EEG monitoring. Particular vigilance is warranted in patients with preoperative seizures or associated brain parenchymal injury, as they carry a higher risk of delayed-onset epilepsy. Standardized antiepileptic drug regimens with timely dose adjustment are recommended to optimize seizure control while minimizing potential adverse effects on neurodevelopment.

#### 5.4.3 Individualized follow-up strategies

Follow-up strategies for pediatric intracranial aneurysms should be individualized. Endovascular treatment requires close monitoring of vascular patency and aneurysm recurrence, while craniotomy necessitates additional assessment of skull healing and cerebral perfusion. Long-term follow-up tailored to age and treatment modality contributes to improved long-term outcomes.

### 5.5 Future treatment prospects

Most currently available flow-diverting devices (FDs) are designed for adult cerebrovascular anatomy, which presents challenges related to device sizing and long-term safety when applied to pediatric patients. Although several studies have reported favorable short-term outcomes with FDs use in pediatric intracranial aneurysms, the overall number of reported cases remains limited (52). In younger children and patients with particularly slender vessels, mismatches between device dimensions and vessel caliber increase the risk of perforator occlusion. Additional concerns include metal surface coverage, endothelialization dynamics, and potential long-term effects on cerebrovascular development. Consequently, the development of pediatric-specific FDs featuring miniaturized profiles, reduced metal coverage, and improved adaptability to immature vascular structures represents an important direction for future innovation (53). In parallel, standardized pediatric anticoagulation and antiplatelet protocols require further refinement to balance thromboembolic prevention with hemorrhagic risk.

Advances in embolization materials also hold promise for improving long-term outcomes in pediatric intracranial aneurysm treatment. Biodegradable embolic materials have been proposed as an alternative to conventional coils, as they may reduce chronic foreign-body reactions and long-term vascular wall irritation after aneurysm occlusion, potentially lowering recurrence rates. However, successful clinical application of such materials depends on precise synchronization between material degradation kinetics and endothelial repair processes, as premature degradation may increase the risk of early recanalization (54).

Multidisciplinary team (MDT) collaboration is essential throughout the diagnosis, treatment, and follow-up of complex pediatric intracranial aneurysms. MDT combined with neurosurgery, interventional neuroradiology, critical care, and rehabilitation helps to optimize clinical outcomes. Meanwhile, artificial intelligence (AI) has shown potential in imaging analysis, aneurysm assessment, and treatment planning, which can further improve diagnostic accuracy and therapeutic efficiency (55).

## 6 Conclusion

Pediatric intracranial aneurysms differ distinctly from adult cases, with a higher proportion of posterior circulation and dissecting aneurysms, rapid progression, and nonspecific symptoms that are easily misdiagnosed. Multimodal imaging (CTA, MRI, DSA) enables early and accurate diagnosis.

Treatment should be individualized based on location and clinical status. Endovascular embolization is preferred for complex posterior circulation aneurysms to avoid traction injury, while selected anterior circulation aneurysms can be safely treated with minimally invasive endovascular techniques. Microsurgery remains necessary for urgent cases with large hematoma or acute brain herniation to achieve rapid decompression. Long-term management requires regular imaging follow-up, especially for dissecting aneurysms, given the relatively high recurrence rate in children.

This study is limited by its retrospective, single-center, small-sample design. However, the four typical cases support the value of individualized strategies. Future research should establish multicenter pediatric aneurysm registries, develop pediatric-specific endovascular devices, and formulate standardized guidelines to improve care for this high-risk population.

## Data Availability

The original contributions presented in the study are included in the article/Supplementary Material, further inquiries can be directed to the corresponding author/s.
